# Bulked Segregant RNA-seq Reveals Differential Expression and SNPs of Candidate Genes Associated with Waterlogging Tolerance in Maize

**DOI:** 10.3389/fpls.2017.01022

**Published:** 2017-06-14

**Authors:** Hewei Du, Jianxiong Zhu, Hang Su, Ming Huang, Hongwei Wang, Shuangcheng Ding, Binglin Zhang, An Luo, Shudong Wei, Xiaohai Tian, Yunbi Xu

**Affiliations:** ^1^Hubei Collaborative Innovation Center for Grain Industry, Yangtze UniversityJingzhou, China; ^2^College of Life Science, Yangtze UniversityJingzhou, China; ^3^Engineering Research Center of Ecology and Agricultural Use of Wetland, Ministry of Education, Yangtze UniversityJingzhou, China; ^4^International Maize and Wheat Improvement Center (CIMMYT)Texcoco, Mexico; ^5^Institute of Crop Science, Chinese Academy of Agricultural SciencesBeijing, China

**Keywords:** maize (*Zea mays* L.), RNA-seq, bulk segregant analysis, waterlogging stress tolerance, SNPs, abiotic stress

## Abstract

Waterlogging has increasingly become one of the major constraints to maize productivity in some maize production zones because it causes serious yield loss. Bulked segregant RNA-seq (BSR-seq) has been widely applied to profile candidate genes and map associated Single Nucleotide Polymorphism (SNP) markers in many species. In this study, 10 waterlogging sensitive and eight tolerant inbred lines were selected from 60 maize inbred lines with waterlogging response determined and preselected by the International Maize and Wheat Improvement Center (CIMMYT) from over 400 tropical maize inbred lines. BSR-seq was performed to identify differentially expressed genes and SNPs associated with waterlogging tolerance. Upon waterlogging stress, 354 and 1094 genes were differentially expressed in the tolerant and sensitive pools, respectively, compared to untreated controls. When tolerant and sensitive pools were compared, 593 genes were differentially expressed under untreated and 431 genes under waterlogged conditions, of which 122 genes overlapped. To validate the BSR-seq results, the expression levels of six genes were determined by qRT-PCR. The qRT-PCR results were consistent with BSR-seq results. Comparison of allelic polymorphism in mRNA sequences between tolerant and sensitive pools revealed 165 (normal condition) and 128 (waterlogged condition) high-probability SNPs. We found 18 overlapping SNPs with genomic positions mapped. Eighteen SNPs were contained in 18 genes, and eight and nine of 18 genes were responsive to waterlogging stress in tolerant and sensitive lines, respectively. Six alleles of the 18 originated from tolerant pool were significantly up-regulated under waterlogging, but not those from sensitive pool. Importantly, one allele (*GRMZM2G055704*) of the six genes was mapped between umc1619 and umc1948 on chromosome 1 where a QTL associated with waterlogging tolerance was identified in a previous research, strongly indicating that *GRMZM2G055704* is a candidate gene responsive to waterlogging. Our research contributes to the knowledge of the molecular mechanism for waterlogging tolerance in maize.

## Introduction

Environmental abiotic stresses, such as drought, submergence or waterlogging, high salinity, and extreme temperatures, severely compromise crop production and productivity (Bray et al., 2000). Submergence and waterlogging have increasingly become one of the major constraints affecting crop yield worldwide. As one of the most important crops, maize is often grown in poorly drained or converted paddy fields, suffering from waterlogging stress in the rain season (Amin et al., 2014). In the monsoon region of Asia, soil flooding during the late spring and early summer is a major source of environmental stress for summer maize (Mano et al., 2006). About 15% of the maize grown in South and Southeast Asia is often subject to waterlogging stress, leading to a 20–30% loss of production each year (Rathore et al., 1998).

Plants have evolved several strategies to adapt to waterlogging or submergence stress. At the molecular level, a large number of genes are induced under waterlogging or submergence conditions that may protect plants from damage caused by waterlogging or submergence. These genes present a valuable tool for improving waterlogging tolerance in maize and other crops. Several important genes conferring submergence tolerance have been identified, including *Sub1A* (submergence 1, from indica cultivar FR13A), *SNORKEL1, SNORKEL2* (from deepwater rice C9285), *RAP2.2* (Related to AP2 2), *HRE1* (hypoxia responsive ERF gene), and *HRE*2 (from *Arabidopsis thaliana*) (Xu et al., 2006; Hattori et al., 2009; Hinz et al., 2010; Licausi et al., 2010). Maize uses a different strategy, including development of adventitious roots, to achieve waterlogging tolerance than rice and *Arabidopsis*, possibly because maize is cultivated in different agroclimatic zones. Maize responds to waterlogging stress through specific alteration of transcription and translation, resulting in morphological adaptation. The above-mentioned genes isolated from rice and *Arabidopsis* may not be effective for improving waterlogging tolerance in maize. Therefore, identification of quantitative trait loci (QTL) and genes associated with waterlogging tolerance in maize is of critical importance for maize improvement.

Several strategies have been applied to identify genes responsible for waterlogging tolerance in maize. A transcriptomic analysis of maize inbred line HZ32 (a selected waterlogging tolerant line) using suppression subtractive hybridization (SSH) identified 63 candidate genes associated with waterlogging tolerance (Zou et al., 2010). Three QTL associated with the ability to form adventitious roots, which are an important characteristic of waterlogging tolerance, were detected (Mano et al., 2005). Three loci, associated with glutamine synthetase, zein, and triosephosphate isomerase, are reported to account for up to 30% of differences in shoot and root dry weights under waterlogging condition (Sérgio et al., 2005). A total of 13, 19, and 23 QTL associated with waterlogging tolerance were identified when subjected to three different periods (3, 6, and 9 days) of waterlogging, respectively (Osman et al., 2013). However, these QTL or candidate genes have still not been applied to improve maize waterlogging tolerance via molecular marker-assisted selection. Therefore, new powerful tools are still needed to identify major QTL for waterlogging tolerance in maize.

Bulked segregant RNA-seq (BSR-Seq), as a new powerful tool, provides an efficient method to rapidly and efficiently map QTL or genes responsive to stresses including waterlogging. Bulked segregant analysis (BSA) can be used to identify markers linked to any specific gene or genomic region using two bulked DNA sample pools. Each pool, or bulk, contains individuals that are identical in a particular trait or genomic region but arbitrary at all unlinked regions (Michelmore et al., 1991). BSA has been extended to bulked sample analysis that can use samples with extreme phenotypes collected from any populations (Zou et al., 2016). The genetic linkage between markers and genes of interest is determined by quantification of allelic frequencies of genetic markers between the two pools (bulks) of plants (Liu et al., 2012). Next-generation sequencing (NGS) provides another powerful means to identify SNPs, the most abundant class of markers, in the genome, in order to reveal candidate mutations in the linkage region that may be causal to the phenotype (Miller et al., 2013). However, for complex traits involving many genes each with minor effect and affected significantly by environments, BSA may not be effective (Zou et al., 2016). With the advancement of NGS, RNA sequencing technology is combined with BSA to develop BSA RNA-seq (BSR-seq). BSR-seq possesses the advantage of BSA and RNA-seq together. It has the full capability to identify differentially expressed genes (DEGs), and also the ability to identify SNPs different between the pools (Wang et al., 2013). BSR-seq has been successfully applied in plants and animals (Liu et al., 2012; Trick et al., 2012; Wang et al., 2013; Yates et al., 2014). For example, 1,255 DEGs and 56,419 SNPs residing on 4,304 unique genes between susceptible and resistant catfish were identified by BSR-seq (Wang et al., 2013). The maize *glossy* mutants exhibit alterations in the accumulation of epicuticular waxes. Two genes, *glossy3* and *glossy13*, were also identified by the BSR-seq approach (Liu et al., 2012; Li et al., 2013). Melon fruit flesh color is associated with *Orange* (*CmOr*) allelic variation. The regulatory network of *CmOr* was identified by BSR-seq (Chayut et al., 2015).

In this study, we applied BSR-seq to identify the DEGs and SNPs in response to waterlogging stress between tolerant and sensitive pools in maize. A total of 18 high-probability SNPs in 18 genes were identified. Six of the 18 genes originated from tolerant lines showed enhanced expression under waterlogging, while not those in sensitive lines, strongly indicating that they may be candidate genes associated with waterlogging tolerance/adaptation in maize. These results will facilitate the study of the molecular mechanism of waterlogging tolerance in maize and the development of waterlogging tolerant varieties via marker-assisted selection.

## Materials and Methods

### Plant Growth and Waterlogging Treatment

Maize seeds were geminated for 2 days in the dark on moist filter paper at room temperature. The germinated seeds were planted in silica sand pots (18 cm × 16 cm), and placed in a greenhouse. Each pot contained six geminated seeds. At three-leaf stage, six pots per genotype were transferred to the pools for waterlogging treatment. In addition, six pots remained untreated (normal condition). The pots were submerged 1∼2 cm under the water surface. All of the waterlogging treatments were performed with three independent biological replicates.

### Phenotypic Analysis

Three traits, including relative shoot height, relative shoot dry weight, and survival rate, were measured as described previously (Du et al., 2016). In brief, plants were subject to waterlogging for 8 days. Three waterlogged and three untreated pots per genotype were chosen for phenotypic analysis. First, the survival rate was determined among 10 waterlogged sensitive and 8 tolerant inbred lines, then seedling height (cm) was measured. After measurement, the shoots were detached, and dried in an oven (65°C) for 3 days. Subsequently, shoot dry weight (g) were determined. A total of 54 seedlings per genotype were measured, and the mean of each trait was calculated by averaging three biological replicates. Shoot height and shoot dry weight of waterlogged plants were compared with those of untreated plants; the results were expressed as percentage of the untreated. One-way ANOVA test was performed to determine the significant differences between tolerant and sensitive inbred lines.

### RNA Isolation

The morphological changes of roots, such as adventitious root formation (ARF), a barrier to radial oxygen loss (ROL), and lysigenous aerenchyma formation, are most important adaptations to flooding or waterlogging condition (Mano et al., 2005; Yamauchi et al., 2011; Abiko et al., 2012). Therefore, roots were selected for BSR-seq in this research. Three pots from each inbred line were used for RNA isolation including untreated plants and those waterlogged for 2 days. Approximately 0.2 g of roots from six seedlings per genotype were detached. The untreated or waterlogged root samples of 10 sensitive inbred lines were pooled; the tolerant pool was generated similarly. Three biological replications were performed for each pool. The root samples were ground to a fine powder in liquid nitrogen. Total RNA samples were isolated using the TRIzol reagent (Invitrogen, Carlsbad, CA, United States). The concentration and purity of extracted RNA was evaluated using the NanoDrop Spectrophotometer (Thermo Scientific-1000), and their integrity was also analyzed using Bioanalyzer (Agilent, 2100). Totally, 12 RNA samples were prepared and submitted to the Beijing Genome Institute (BGI) for sequencing.

### Sequencing and Transcriptome Construction

The 12 RNA pools (tolerant and sensitive × untreated and 2-day waterlogged × three biological replicates) were RNA-sequenced at BGI. Twelve libraries were constructed, and paired-end sequencing was performed according to manufacturer’s instructions (Illumina, San Diego, CA, United States). Briefly, Poly-A RNA containing mRNA was enriched using poly-T oligo-attached magnetic beads and fragmented. Second-strand cDNA was synthesized using random hexamer primers, then purified, end-repaired, poly-A tailed, and adaptor ligased. The cDNA pools were loaded to Illumina Hiseq2000 sequencer (Illumina, United States) for sequencing. Library construction and RNA-seq were conducted at BGI.

A large number of raw reads were generated from sequencing machine. The quality evaluation, filtering, processing and alignment of the sequence reads were performed according to previous reports (Li et al., 2008; Cock et al., 2010; Chatterjee et al., 2012). In brief, the quality of reads was validated using FastQC (version 0.11.3) (Cock et al., 2010). Reads containing more than 5% unknown nucleotides and low-quality reads (≤20% of the bases with a quality score ≤ 10) were discarded. The adaptor contamination was removed using Clean adaptors software (Chatterjee et al., 2012). The clean reads were aligned to the B73 reference genome (ZmB73_RefGen_V3) using TopHt V2.0.6 according to Li et al. (2008). The genome alignment and contig assembly was carried out by BGI. These data have been uploaded to the NCBI SRA database (Bioproject: PRJNA387650).

### Annotation and Gene Ontology (GO) Analysis

Annotation of transcript tags was conducted. The BLASTX was performed using the contigs against the MaizeGDB database with the maximum *E*-value of 1e-15. The best match per BLAST search was used to annotate the transcript tags. The contigs with no matches were classified as “predicted” or “uncharacterized” annotation. GO analysis was performed using GO Term Finder to describe the biology of a gene product, such as molecular function, biological process, and cellular component^1^.

### Metabolic Pathway and Significance of Digital Gene Expression Profiles

Identification of significantly DEGs was conducted according to Audic and Claverie (1997), and Reiner et al. (2003). In brief, genes differentially expressed between different pools were detected from the variations in the counts of their cognate sequence tags. The statistical significance of differential expression was determined using multiple testing combined with false discovery rate (FDR). In this study, genes with greater than two-fold expression levels and FDR ≤ 0.001 (*P*-value < 0.05) were classified as significant DEGs. The metabolic pathway annotation was carried out using Blastall software against the KEGG (Kyoto Encyclopedia of Genes and Genomes) database (Mckenna et al., 2010).

### qRT-PCR

The roots at different time points of waterlogging were collected from six seedlings, and the total RNA samples were isolated using the TRIzol reagent (Invitrogen, Carlsbad, CA, United States). The synthesis of first strand cDNA and qRT-PCR was performed according to Du et al. (2016). In brief, the first stand cDNA was synthesized using SuperScript-II reverse transcriptase (Invitrogen). The *actin1* gene (*GRMZM2G126010*) was chosen as an internal control to normalize the expression data. The primer sequences of genes used in this study are listed **Supplementary Table S1**. The expression level was determined using the dd2ˆC value obtained from real-time PCR. Real-time PCR was carried out using the SYBR real-time PCR kit (Takara Japan) with IQTM SYBR^®^ Green Supermixture according to the manufacturer’s instruction (Bio-Rad, United States). The reaction conditions were as follows: 94°C for 1 min, followed by 40 cycles of 95°C for 10 s, 55°C for 10 s, and 72°C for 15 s.

### SNPs Calling and Filtration

The Genome Analysis Toolkit (GATK) software was employed to identify SNPs (Mckenna et al., 2010). All the clean reads were used to identify SNPs according to Liu et al. (2012). In brief, validated SNP site must have two and only two SNP-types. Reads from these two SNP-types must account for ≥90% of the total reads that align to this SNP site. Each SNP-type must have ≥3 reads and the reads account for ≥20% of the total reads on that SNP site (Liu et al., 2012). For each SNP, Bayes’ theorem was approached to estimate the linkage probability between a SNP and the causal gene (Liu et al., 2012).

## Results

### Waterlogging Tolerant and Sensitive Genotypes Identified

**FIGURE 1:**
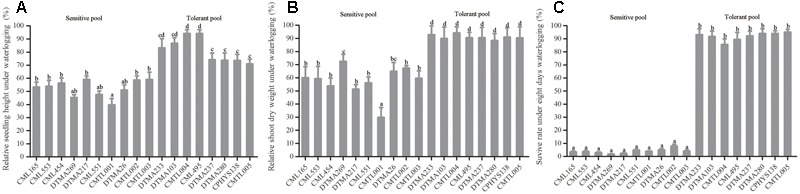
Differences in plant growth traits between tolerant and sensitive inbred groups upon waterlogging stress. **(A)** Relative shoot height, **(B)** relative shoot dry weight, **(C)** Survival rate. All experiments included three biological replications. Six germinated seeds were planted into each pot. For each inbred line, three pots containing 18 seedlings at the three-leaf stage were subjected to waterlogging treatment. After 8 days of waterlogging treatment, traits including survival rate and shoot height were measured. After measurement, shoots and roots were excised, transferred into envelope, and placed in an oven (65°C) for 3 days; shoot weight was then measured. Shoot height and shoot dry weight of waterlogged plants were compared to those of untreated plants. Each bar (in cm or g) represents the mean value ± SD of three independent analyses. One-way ANOVA test was performed to reveal the significance between tolerant and sensitive groups under waterlogging treatment. Among these lines, the different letters on the bars mean significant difference.

Sixty tropical maize inbred lines were determined and selected for waterlogging tolerance by CIMMYT from over 400 tropical maize inbred lines (Yunbi Xu, personal communication). In this study, the sixty maize inbred lines, were retested by subjecting them to waterlogging treatment in the greenhouse. Three traits were chosen as selection criteria to distinguish tolerant from sensitive lines, including relative shoot height, relative shoot dry weight, and survival rate. These traits were measured for each line after waterlogging treatment for 8 days. Another set, was left untreated as control. Each trait was compared between waterlogged and untreated maize lines. According to these criteria, eight inbred lines displayed enhanced tolerance to waterlogging under 8-day waterlogging. By contrast, ten inbred lines were sensitive to waterlogging. Therefore, we chose 8 tolerant and 10 sensitive inbred lines to form the tolerant and sensitive pools, respectively. The phenotype of these inbred lines was analyzed. Under waterlogging, the shoot height decreased both in tolerant and sensitive inbred groups. The average relative shoot height of the sensitive group was reduced more than 40% (**Figure 1A**), indicating serious suppression on shoot elongation by waterlogging. Especially, relative shoot height of line CMTL001 was reduced to approximately 40% (**Figure 1A**). The average relative shoot height of the tolerant group is only reduced to 70% (**Figure 1A**), significantly higher than that of the sensitive group. Most significantly, lines CMTL004 and CML495 retained relative shoot heights at 90% (**Figure 1A**).

Relative shoot dry weight also decreased under waterlogging in tolerant and sensitive groups compared with untreated controls. However, the sensitive group was affected much greater than the tolerant group. The average relative shoot dry weight of the sensitive group was reduced to less than 73%, compared to the control. Especially, line CMTL001 measured at only 30%, indicating severe shoot growth retardation. However, the tolerant group retained the average relative shoot mass at 88% (**Figure 1B**), indicating relatively well-developed shoots.

Survival rate was the most important criterion to measure tolerance in maize. The sensitive group showed less than 5% survival rate under 8 days of waterlogging treatment (**Figure 1C**). In contrast, the tolerant group showed more than 85% survival rate (**Figure 1C**). Therefore, the eight inbred lines in the tolerant group were more tolerant to waterlogging than those in the sensitive group. In addition, the tolerant group grew much stronger than the sensitive group after waterlogging treatment for 8 days (**Supplementary Figure S1**). For further study, therefore, we selected 10 sensitive and 8 tolerant lines from these groups for BSR-seq based on our results and those reported by CIMMYT.

### Sequences Assembled and Analyzed

Seedlings of 8 tolerant and 10 sensitive inbred lines grown in the greenhouse at three-leaf stage were subjected to waterlogging treatment for 2 days (Zhang et al., 2008); a parallel set was left untreated as control. RNA samples were extracted from these pools and RNA-seq was conducted with three biological replications using Illumina sequencing at Shenzhen BGI. Approximately 4.5 billion base pairs from 30 million reads with an average of 150 bp were generated from each pool (**Table 1**). For the sensitive pool, approximately 4.6 billion base pairs (bps) from 30.65 million reads were generated for waterlogged and 4.58 billion bps from 30.55 million reads for the untreated. For the tolerant pool, 4.57 billion bps from 30.49 million reads were generated for the waterlogged and 4.54 billion bps 30.28 million reads for the untreated. For the sensitive pool, 57.08% of untreated and 56.77% of waterlogged reads were mapped to the genome. For the tolerant pool, 57.80% untreated and 58.16% waterlogged reads were mapped to the genome; 67.34% untreated and 67.93% waterlogged reads mapped to genes. Each pool contained approximately 40K expressed transcripts, either untreated or waterlogged (**Table 1**).

**Table 1:** Summary of RNA-seq results of tolerant and sensitive pools under waterlogged or normal condition.

	Condition	Replicates	Clean reads		Total base pairs		Genome map rate		Gene map rate		Expressed gene	
Sample Pool			Number	Mean (Million)	Number	Mean (Billion)	Percentage (%)	Mean (%)	Percentage (%)	Mean (%)	Number	Mean
sensitive pool		1	30243612		4536541800		55.85		69.06		31582	
	Normal	2	30582784	30.55 ± 0.29	4587417600	4.58 ± 0.04	57.40	57.08 ± 1.10	68.07	68.04 ± 1.03	31527	31550 ± 28
		3	30827478		4624121700		57.98		67.00		31541	
		1	30628326		4594248900		56.99		66.37		31391	
	Waterlogging	2	30653618	30.65 ± 0.22	4598042700	4.60 ± 0.03	56.67	56.77 ± 0.19	65.43	65.50 ± 0.84	30642	31062 ± 382
		3	30671452		4600717800		56.64		64.7		31154	
tolerant pool		1	30714780		4607217000		58.23		68.41		30907	
	Normal	2	29372898	30.28 ± 0.79	4405934700	4.54 ± 0.12	57.09	57.80 ± 0.62	66.16	67.34 ± 1.13	31443	31201 ± 271
		3	30767808		4615171200		58.07		67.44		31255	
		1	30333212		4549981800		57.61		68.04		31480	
	Waterlogging	2	30386534	30.49 ± 0.23	4557980100	4.57 ± 0.04	58.31	58.16 ± 0.49	67.67	67.93 ± 0.23	31077	31279 ± 201
		3	30762462		4614369300		58.56		68.08		31280	

### Genes Differentially Expressed upon Waterlogging Stress

The transcriptomic profiles of tolerant and sensitive pools, untreated or waterlogged, were built by comparing their gene expression levels based on RPKM (reads per kilobase of exon model per million mapped reads). For the tolerant pool, 354 genes were differentially expressed (>2-fold) upon waterlogging (**Table 2**), among which 173 genes were down-regulated while 181 up-regulated (**Table 2**). Among the 173 down-regulated genes, one gene was down-regulated at over 1000×, two genes at over 100×, and 16 genes at 10–100×; among the 181 genes up-regulated, two were up-regulated at over 1000×, six at over 100×, and 37 genes at 10–100× (**Table 2**).

**Table 2:** Comparison of gene expression between normal and waterlogging in tolerant or sensitive pool.

Differentially expressed genes in sensitive pool between waterlogged and untreated control	1094	Differentially expressed genes in tolerant pool between waterlogged and untreated control	354
Gene expressed higher in sensitive pool	555	Gene expressed higher in tolerant pool	181
>1000-fold	3	>1000-fold	2
100- to 1000-fold	26	100- to 1000-fold	6
10- to 100-fold	126	10- to 100-fold	37
2- to 10-fold	400	2- to 10-fold	136
Genes expressed lower in sensitive pool	539	Genes expressed lower in tolerant pool	173
>1000-fold	2	>1000-fold	1
100- to 1000-fold	12	100- to 1000-fold	2
10- to 100-fold	79	10- to 100-fold	16
2- to 10-fold	446	2- to 10-fold	154

The sensitive pool showed a larger number (1094 genes) of DEGs upon waterlogging stress. Among these genes, 539 genes were down-regulated (>2-fold) and 555 were up-regulated (**Table 2**). Among the 539 down-regulated genes, two down-regulated at over 1000×, 12 at over 100×, and 79 genes at 10–100×; among the 555 up-regulated genes, three were up-regulated at over 1000×, 26 at over 100×, and 126 genes at 10–100× (**Table 2**).

### Comparative Gene Expression in Tolerant and Sensitive Pools under Waterlogging Stress

The DEGs between tolerant and sensitive pools were also determined. Without waterlogging treatment, 593 genes displayed significantly differential expression between tolerant and sensitive pools, with 195 genes down-regulated and 398 genes up-regulated in the tolerant pool compared to the sensitive pool (**Table 3**). Among the 195 down-regulated genes, four were down-regulated at over 1000×, 18 at over 100×, and 25 at 10–100×. Among the 388 up-regulated genes, three were up-regulated at over 1000×, 19 at 100–1000×, and 49 at 10–100× (**Table 3**).

**Table 3:** Comparison of gene expression between tolerant and sensitive pools under waterlogging or normal condition.

Differentially expressed genes between tolerant and sensitive pools at normal condition	593	Differentially expressed genes between tolerant and sensitive pools under waterlogging stress	431
Gene expressed higher	398	Gene expressed higher	179
>1000-fold	3	>1000-fold	5
100- to 1000-fold	19	100- to 1000-fold	23
10- to 100-fold	49	10- to 100 fold	39
2- to 10-fold	327	2- to 10-fold	112
Genes expressed lower	195	Genes expressed lower	252
>1000-fold	4	>1000-fold	2
100- to 1000-fold	18	100- to 1000-fold	29
10- to 100-fold	25	10- to 100-fold	48
2- to 10-fold	148	2- to 10-fold	173

Less DEGs were identified with waterlogging treatment between tolerant and sensitive pools. A total of 431 genes were significantly differentially expressed between the tolerant and sensitive pools under waterlogging; among them, 252 genes were down-regulated and 179 genes up-regulated (**Table 3**). Among the down-regulated genes, two were down-regulated at 1000×, 29 at 100–1000×, and 48 genes at 10–100×. Among the up-regulated genes, five were up-regulated at over 1000×, 23 at 100–1000× and 39 at 10–100× (**Table 3**).

Among the 593 and 431 DEGs identified in untreated and waterlogged samples, respectively, 122 genes overlapped, among which, 11 genes displayed opposite expression levels between waterlogged and normal conditions. Ten genes (*GRMZM2G116520, GRMZM2G106445, GRMZM2G305362, GRMZM2G076972, GRMZM2G374971, GRMZM2G098875, ZEAMMB73_943532, GRMZM2G127418, GRMZM2G053503*, and *GRMZM2G169240*) were down-regulated under normal condition whereas up-regulated under waterlogged condition in the tolerant pool. Only one gene (*GRMZM2G097229*) was up-regulated under untreated whereas down-regulated under waterlogged condition in the tolerant pool. For the remaining 111 genes, 45 genes were down-regulated under untreated and 66 genes up-regulated under waterlogged condition in tolerant pool (**Supplementary Table S2**).

### RNA-seq Results Validated by qRT-PCR

**FIGURE 2:**
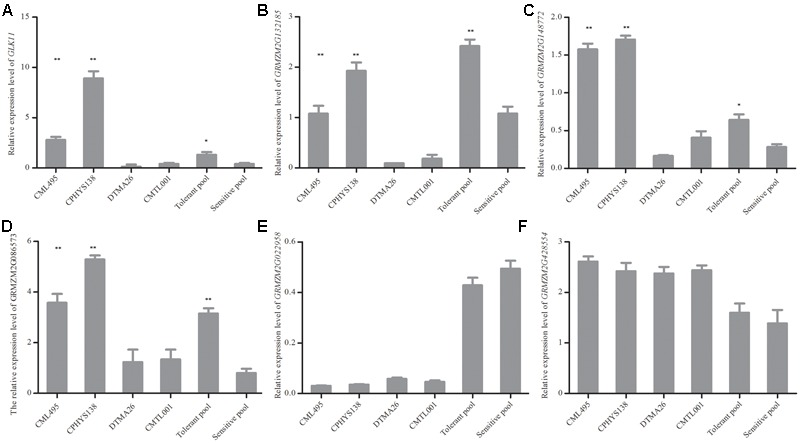
Changes of expression levels for six genes were revealed by RNA-seq and validated by qRT-PCR. **(A–F)** Relative expression level of *GLK11, GRMZM2G132185, GRMZM2G148772, GRMZM2G086573, GRMZM2G022958*, and *GRMZM2G428554*, respectively. Approximately 0.2 g of roots were excised from six seedlings and used for RNA isolation. Six genes were selected based on RNA-seq results, and their expression levels were validated by qRT-PCR. All experiments included three biological replications. Each bar represents the fold change in expression level of the gene by comparing waterlogged with normal conditions. Student’s *t*-test was performed. ^∗^Indicates *p* < 0.05; ^∗∗^indicates *p* < 0.01.

**FIGURE 3:**
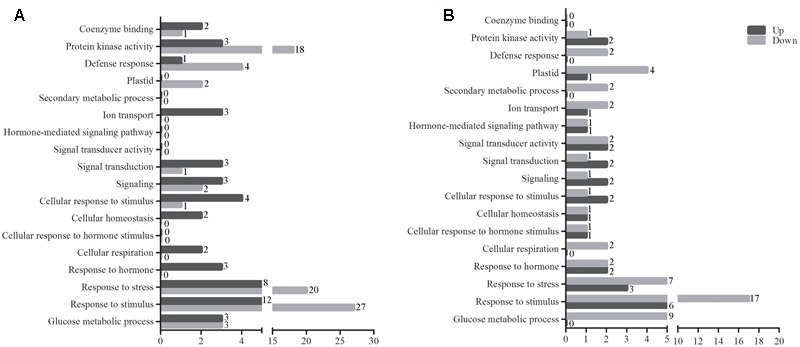
The GO term analysis of DEGs between tolerant and sensitive pools. GO term analysis of significant DEGs between tolerant and sensitive pools at normal condition **(A)** and under waterlogging stress **(B)**. GO analysis was performed using GO Term Finder. The red and blue bars represent the numbers of up- and down-regulated genes between tolerant and sensitive pools, respectively. The GO terms are shown on the *y*-axis and the number of significant DEGs is at the end of each bar.

To confirm the results of RNA-seq, qRT-PCR was performed. Six genes (*GLK11* (Maizegdb: *AC233960.1*), *GRMZM2G132185, GRMZM2G148772, GRMZM2G086573, GRMZM2G022958*, and *GRMZM2G428554*) were investigated in two tolerant (CML495, CPHYS138) and two sensitive (DTMA26, CMTL001) inbred lines. Under waterlogging, their relative expression levels (compared to those under normal condition) were determined by qRT-PCR (**Figure 2**). According to the RNA-seq data, three genes (*GLK11, GRMZM2G132185*, and *GRMZM2G086573*) were expressed significantly higher in tolerant pool than sensitive pool (**Figures 2A,B,D**). *GRMZM2G148772* were down-regulated both in tolerant and sensitive pools, but the expression level of *GRMZM2G148772* in tolerant pool was significantly higher than that of sensitive pool (**Figure 3**). *GRMZM2G022958* and *GRMZM2G428554* did not differentially express between tolerant and sensitive pools. The qRT-PCR was performed to validate the RNA-seq data. Upon waterlogging treatment, *GLK11, GRMZM2G132185*, and *GRMZM2G086573* were up-regulated in two tolerant lines (CML495 and CPHYS138), but down-regulated in two sensitive lines (DTMA26 and CMTL001) (**Figures 2A,B,D**). *GRMZM2G148772* was up-regulated in two tolerant lines (CML495 and CPHYS138), but down-regulated in two sensitive lines (DTMA26 and CMTL001). *GRMZM2G022958* was down-regulated in both tolerant and sensitive lines, while *GRMZM2G428554* was up-regulated. Neither of *GRMZM2G022958* and *GRMZM2G428554* differently expressed between tolerant and sensitive lines. Except for enhanced expression of *GRMZM2G148772* in tolerant lines under waterlogging, the rest results of qRT-PCR were consistent with that of RNA-seq data. Overall, our qRT-PCR results generally confirmed our RNA-seq results.

### Functional and Metabolic Pathways Revealed by Gene Ontology (GO) Analysis

Gene ontology analysis was subsequently carried out for the DEGs using GO Term Finder^2^. **Figure 3** shows the numbers of genes differentially expressed between tolerant and sensitive pools under various GO items. Protein kinases, such as mitogen-activated protein kinases (MAPKs), can respond to various extracellular stimuli and act as key signaling components to activate various cellular activities (Rohila and Yang, 2007). Under protein kinase activity, 18 genes were up-regulated and 3 down-regulated under normal condition (**Figure 3A** and **Supplementary Table S2**); under waterlogged condition, two genes were up-regulated and one down-regulated (**Figure 3B** and **Supplementary Table S2**). Under the response to stress and stimulus GO term, 28 and 39 genes were identified under normal condition, respectively; 20 and 27 of these genes were up-regulated, respectively (**Figure 3A** and **Supplementary Table S3**). No DEGs under secondary metabolic process, hormone-mediated signaling pathway, signal transducer activity, or cellular response to hormone stimulus GO terms were identified under normal condition (**Figure 3A**). In contrast, upon waterlogging treatment, two genes belonging to the secondary metabolic process GO term were down-regulated. In addition, two genes in hormone-mediated signaling pathway, four in signal transducer activity, and two in cellular response to hormone stimulus GO terms were differentially expressed (**Figure 3B** and **Supplementary Table S3**). Waterlogging treatment resulted in more DEGs belonging to the GO terms of plastid, secondary metabolic process, hormone-mediated signaling pathway, signal transducer activity, cellular response to hormone stimulus, response to hormone, and glucose metabolic process than normal condition (**Figure 3B** and **Supplementary Table S3**). Signaling and signal transduction are critical steps to adaptation to abiotic stresses in plants. Four DEGs fall in the GO terms of signal transducer activity, with two genes up-regulated. Three DEGs fall in signal transduction and signaling GO terms, with two genes up-regulated (**Figure 3B** and **Supplementary Table S3**).

Interestingly, several of the genes overlapped in different GO terms. Under normal condition, one up-regulated gene falls in signal transduction, cell response to stimulus, response to stimulus, and signaling GO terms (**Figure 3A**); three down-regulated gene fall in signal transduction, signaling, response to stimulus, cell response to stimulus (**Figure 3A** and **Supplementary Table S3**). Under waterlogging treatment, one up-regulated gene falls in the hormone-mediated signaling pathway, response to hormone, signal transduction, cellular response to stimulus, and signaling (**Figure 3B** and **Supplementary Table S3**). In addition, three up-regulated and seven down-regulated genes fall in both response to stress and response to stimulus (**Figure 3B** and **Supplementary Table S3**).

Importantly, DEGs identified between waterlogged and untreated controls also fall into multiple GO terms. A gene differentially expressed between waterlogged and untreated control falls in defense response (*NM_001147821.1*), cellular homeostasis (*XR_558874.1*), cellular respiration (*XR_557205.1*), and response to stimulus (*XP_008675697.1*) (**Figure 3** and **Supplementary Table S3**), respectively. Interestingly, one gene (*GRMZM2G374971*) was up-regulated in both response to stimulus and defense response under normal condition, whereas down-regulated upon waterlogging treatment (**Figure 3** and **Supplementary Table S3**).

### SNPs Associated with Waterlogging Stress

**FIGURE 4:**
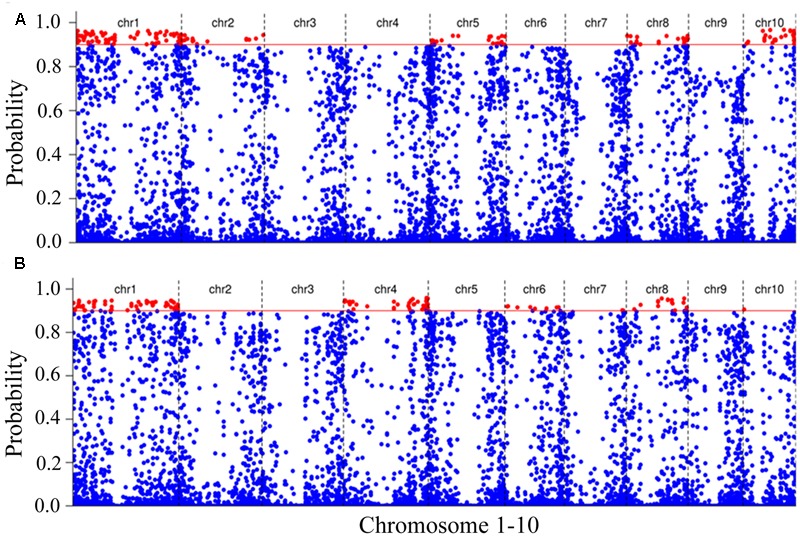
Putative SNPs associated with waterlogging stress. The polymorphic SNPs between tolerant and sensitive pools under normal condition **(A)** and waterlogging treatment **(B)**. The polymorphic SNPs were identified by comparing RNA sequences of tolerant and sensitive pools. The linkage probability of each SNP marker associated with the causal gene was obtained from a Bayesian BSA analysis. The physical position of each SNP marker (*x*-axis) was plotted vs. the linkage probability (*y*-axis). The red solid line indicates the threshold of linkage probability (0.9).

In order to map the genes associated with waterlogging stress adaptation, we identified the SNPs present in RNAs contained in the RNA-seq data between the tolerant and sensitive pools. A total of 114,580 high-confidence SNPs between the tolerant and sensitive pools were identified under normal condition (**Supplementary Table S4**), and 114,464 SNPs identified under waterlogging treatment (**Supplementary Table S5**). To identify SNPs linked to genes responsive to waterlogging stress, we used an empirical Bayesian approach to estimate the linkage probability between a SNP and the causal gene. The linkage probability of each SNP was plotted against its physical coordinate in the B73 genome as shown in **Figure 4**. Under normal condition, there were 165 SNPs with high probabilities (>0.9) between the tolerant and sensitive pools (**Supplementary Table S6**). There were 71, 17, 22, 25, and 20 high-probability SNPs on chromosomes 1, 2, 5, 8, and 10, respectively (**Figure 4A**). Many genes contained multiple SNPs: 17 genes contained two polymorphic SNPs, five genes (*GRMZM2G083253, GRMZM2G088765, GRMZM2G115456, GRMZM2G8259788*, and *GRMZM2G833066*) contained three SNPs, and two genes (*GRMZM2G079777* and *GRMZM2G143071*) contained four SNPs (**Supplementary Table S5**). Six genes (*GRMZM2G102760, GRMZM2G434203, GRMZM2G154870, GRMZM2G400714, GRMZM2G080992*, and *GRMZM5G822237*) displayed both SNP and differential expression levels between the tolerant and sensitive pools under normal condition, and have been mapped on chromosomes (**Figure 4**).

**FIGURE 5:**
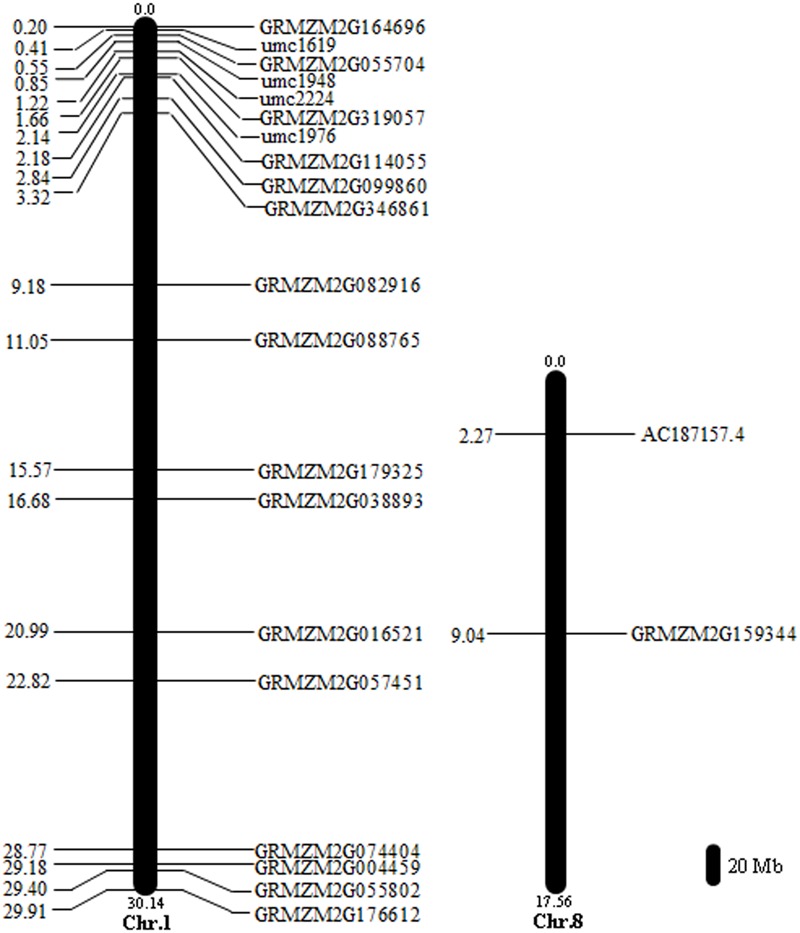
Locations of 18 genes containing significant SNPs on the maize chromosomes. The scale represents a 20 Mb chromosomal distance. The numbers in distance are in the scale of Mb. Chromosome numbers are indicted at the bottom end of each chromosome.

We identified 128 SNPs between tolerant and sensitive pools under waterlogged condition, and localized them on chromosomes 1 (58 SNPs), 4 (39), 5 (1), 6 (9), 7 (2), 8 (18), and 10 (1) (**Figure 4B**), respectively, that exhibited high-linkage probability (>0.9) with genes responsive to waterlogging stress. Fifteen genes contained two SNPs; five genes (*GRMZM2G038893, GRMZM2G063917, GRMZM2G088765, GRMZM2G159344*, and *GRMZM2G378907*) contained three SNPs (**Supplementary Table S7**). Three genes (*GRMZM2G022958, GRMZM2G039009*, and *GRMZM2G428554*) differentially expressed between tolerant and sensitive pools under waterlogging also exhibited SNPs polymorphism, and were mapped on chromosomes (**Figure 4**). More importantly, 18 SNPs overlapped between the 165 and 128 SNPs and contained in 18 genes (*AC187157.4, GRMZM2G004459, GRMZM2G016521, GRMZM2G038893, GRMZM2G055704, GRMZM2G055802, GRMZM2G057451, GRMZM2G074404, GRMZM2G082916, GRMZM2G088765, GRMZM2G099860, GRMZM2G114055, GRMZM2G159344, GRMZM2G164696, GRMZM2G176612, GRMZM2G179325, GRMZM2G319057*, and *GRMZM2G346861*). Among these 18 genes, two (*AC187157.4* and *GRMZM2G159344*) were localized on chromosome 8, and the other 16 genes on chromosome 1 (**Figure 5**).

The putative functions of these 18 genes were annotated (**Supplementary Table S8**). *GRMZM2G179325* encodes a GRAS [gibberellic acid insensitive (GAI), repressor of ga1-3 (RGA), and scarecrow (SCR)] transcriptional factor. *GRMZM2G055704* encodes a detoxification superfamily protein, a heavy metal transporter. *GRMZM2G114055* encodes a protein belonging to the non-phototropic hypocotyl 3 (NPH3) family that responds to environmental or endogenous signals. *AC187157.4* contains an AP2 domain and belongs to AP2/ERF superfamily. These gene families have been well documented to be responsive to abiotic stress in plants (Xu et al., 2006, 2015; Furutani et al., 2011; Shabala et al., 2014), indicating that four (*GRMZM2G179325, GRMZM2G055704, GRMZM2G114055*, and *AC187157.4*) of the 18 genes may represent known gene families associated with waterlogging stress. The rest may belong to novel genes families involved in waterlogging stress in maize.

### Genes with Significantly SNP Response to Waterlogging Stress

**FIGURE 6:**
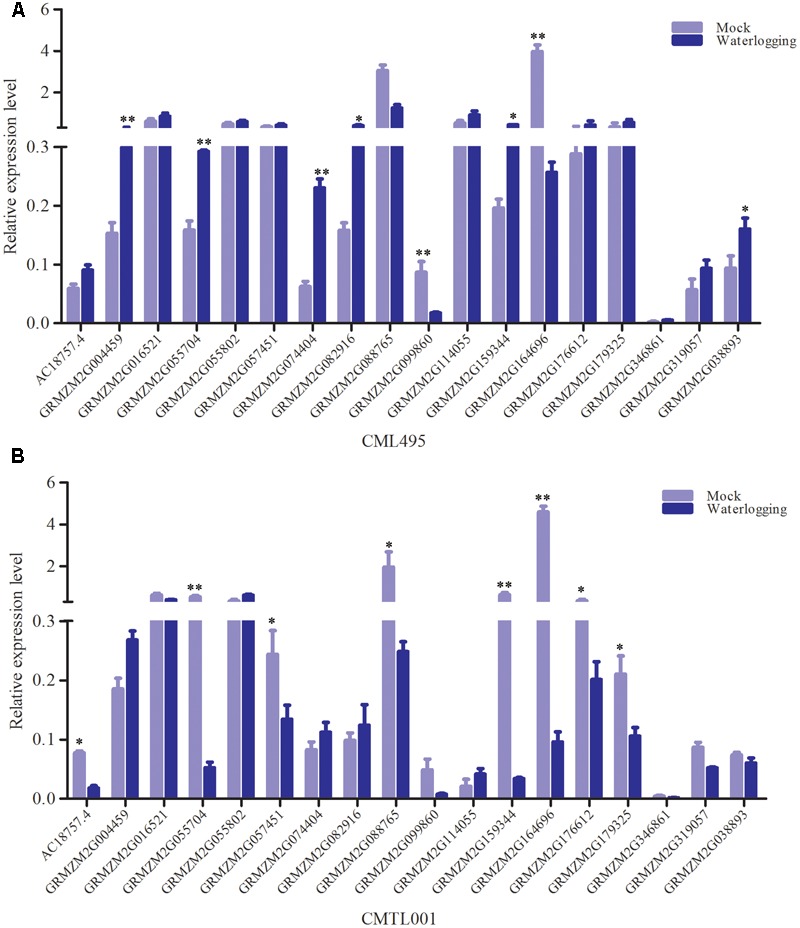
The relative expression level of 18 genes with high-probability under waterlogging stress. **(A)** The expression level in tolerant line (CML495). **(B)** The expression level in sensitive line (CMTL001). Approximately 0.2 g of roots were excised from six seedlings and used for RNA isolation. A total of 18 genes with significant SNP overlapped between tolerant and sensitive pool under waterlogging and normal conditions, and their expression levels were validated by qRT-PCR. All experiments included three biological replications. Each bar represents the expression level of gene. Student’s *t*-test was performed. ^∗^Indicates *p* < 0.05; ^∗∗^indicates *p* < 0.01.

Between sensitive and tolerant pools, a total of 18 genes with high probabilities SNP overlapped under waterlogging and normal conditions, indicating that these genes were responsive to waterlogging stress. However, according to RNA-seq data, these genes did not show differential expression between the pools under waterlogging. To confirm these genes responsive to waterlogging stress, qRT-PCR was performed. One tolerant (CML495) and sensitive inbred lines (CMTL001) were chosen, and subjected to waterlogging at three-leaf stage. Under waterlogging stress, the qRT-PCR results showed that *GRMZM2G004459* was significantly up-regulated in both CML495 and CMTL001 (**Figure 6**). *GRMZM2G074404, GRMZM2G082916*, and *GRMZM2G038893* were significantly up-regulated in CML495, a tolerant line, while not in CMTL001, a sensitive line (**Figure 6**). Two genes, (*GRMZM2G099860* and *GRMZM2G164696*), were significantly down-regulated in both CML495 and CMTL001 (**Figure 6**). In addition, the expression level of six genes (*GRMZM2G057451, GRMZM2G055704, GRMZM2G159344, GRMZM2G088765, GRMZM2G176612*, and *GRMZM2G179325*) significantly decreased in CMTL001 (**Figure 6B**), a sensitive line, but not in CML495 (**Figure 6A**). Among 18 genes, eight and nine genes were up- or down-regulated in CML495 and CMTL001 under waterlogging, respectively. Above results confirm that most of genes with high-probability SNP were responsive to waterlogging stress, indicating that these genes may be strong candidate genes associated with waterlogging tolerance in maize.

### Genes with High-Probability SNP Originated from Tolerant Pool Were Up-regulated

As inbred lines in the tolerant pool is more tolerant to waterlogging than those of sensitive pool, an interesting question to ask is which allele was induced to express under waterlogging. A total of 165 and 128 high probabilities SNPs between tolerance and sensitive pools were identified under normal and waterlogging conditions, respectively, 18 of which overlapped, and were located on 18 genes. Six of the 18 genes (*GRMZM2G016521, GRMZM2G055704, GRMZM2G057451, GRMZM2G159344, GRMZM2G176612*, and *GRMZM2G038893*) originated from sensitive pool with high-probability SNPs consistent with reference genome (B73, a waterlogging sensitive inbred line) (**Supplementary Tables S6, S7**), and were down-regulated under waterlogging stress (**Figure 6**). However, their alleles originated from tolerant pool displayed differential SNPs (**Supplementary Tables S6, S7**), and were up-regulated under waterlogging (**Figure 6**). Therefore, some alleles originated from tolerant pool were preferentially induced to express under waterlogging.

## Discussion

### BSR-seq Is an Idea Tool to Identify Candidate Genes Responsive to Waterlogging

Although the maizeGDB and NCBI databases contain a large number of resources, including ESTs and gene expression, additional resources are needed for identification and mapping of the genes involved in waterlogging tolerance in maize. In this study, NGS RNA-seq was conducted to analyze the DEGs identified between waterlogging tolerant and sensitive pools in maize. These data not only allowed identification of the genes differentially expressed between the pools, but also allowed mapping the linked genes on the whole genome and quantifying allele-specific gene expression.

Waterlogging stress at early growth stages is most destructive to maize plants because maize is highly susceptible to waterlogging at the early vegetative seedling (V2) stage (Zaidi et al., 2004). Therefore, identification and isolation of genes responsive to waterlogging at the V2 stage would be most useful for improving waterlogging tolerance in maize. Maize is cultivated in diverse agro-climatic zones extending from the subtropical to the cooler temperate regions (Amin et al., 2014), indicating that maize contains extensive genetic diversity to adapt to a series of environmental challenges, including waterlogging. Furthermore, tolerant and sensitive maize germplasms are available, allowing the selection of 10 sensitive and eight tolerant inbred lines from over 400 maize inbred lines. Pooling strategy was applied to reduce genetic differences in non-targeted traits within and between pools, allowing us to focus on waterlogging tolerance. RNA-seq was subsequently applied to identify genes differentially expressed between tolerant and sensitive pools. This BSR-seq approach has been widely used in other studies, including studies of metabolic and cellular processes associated with β–carotene accumulation in melon (Chayut et al., 2015), fine-mapping and cloning of genes in diploid wheat (Trick et al., 2012), analysis of genetic basis of drought tolerance in red clover (Yates et al., 2014), and gene mapping in maize (Liu et al., 2012). Here we identified approximately 32,000 genes expressed in the roots, and among them, 431 genes (1.3%) were expressed differentially between tolerant and sensitive pools under waterlogging. Because only approximately 1% of the genes were differentially expressed upon waterlogging treatment, expression of these genes likely represents a response to waterlogging stress rather than an overall genetic difference between the tolerant and sensitive lines, which should be much greater.

Previous researches have applied several strategies to identify and map genes and QTL involved in waterlogging response in maize. For example, 55 QTL associated with waterlogging tolerance in maize were identified, and seven important QTL were mapped onto five linkage groups using a F_2_ population (Osman et al., 2013). Reverse Northern analysis of a set of 768 cDNA clones identified from a SSH library revealed that a large number of genes were up-regulated by waterlogging stress in maize, and 63 of them were co-localized with reported QTL (Zou et al., 2010). A genome-wide association study (GWAS) has been performed using 144 maize inbred lines. Several traits were measured, including length, fresh and dry weights of roots and shoots under waterlogging and normal conditions. Forty seven SNPs were significantly associated with six traits. Among these 47 SNPs, 33 SNPs matched previously reported ones in waterlogging-related traits (Zhang et al., 2013). Here we applied the new BSR-seq strategy and found that 431 genes were differentially expressed in tolerant pool compared to the sensitive pool when subject to waterlogging. Identification of these genes lays a cornerstone to the isolation of candidate genes involved in adaptation to waterlogging.

### The Candidate Genes Responsive to Waterlogging

The candidate genes responsive to waterlogging stress should be correlated with their differential mRNA levels and SNPs polymorphic between the tolerant and sensitive pools. RNA-seq reads can be used to identify DEGs and mine allelic polymorphisms across the whole genome (Liu et al., 2012). Based on this hypothesis, BSR-seq was performed to analyze DEGs under normal and waterlogged conditions with 593 and 431 genes identified (**Table 3**). In addition, 165 and 128 high probabilities SNPs were also identified (**Supplementary Tables S5, S6**), which 18 SNPs were overlapped. Eighteen SNPs were contained in 18 genes, respectively, and were mapped on chromosome. Six of 18 genes originated from sensitive line (CMTL001) were significantly down-regulated under waterlogging stress, while not in tolerant line (CML495). Six genes (*GRMZM2G057451, GRMZM2G055704, GRMZM2G159344, GRMZM2G088765, GRMZM2G176612*, and *GRMZM2G179325*) displayed DEGs and SNP between tolerant and sensitive pool under waterlogging treatment, which strong indicated that them were candidate genes responsive to waterlogging.

In a previous research, many QTL associated with waterlogging stress have been mined (Osman et al., 2013), which showed that those QTL were located in umc1619 ∼ umc1948 on chromosome 1 (Osman et al., 2013). In our results, one of six differentially expressed and polymorphic SNP genes (*GRMZM2G055704*) was significantly up-regulated in tolerant line (CML495), while significantly down-regulated in sensitive line (CMTL001). Importantly, *GRMZM2G055704* was mapped in umc1619 ∼ umc1948 (**Figure 5**), indicating that our results are consistent with the previous report. Therefore, *GRMZM2G055704* may be a candidate gene responsive to waterlogging. Meanwhile, the rest are novel genes responsive to waterlogging stress, which were identified in this report will facilitate the understanding of the molecular mechanism of waterlogging response in maize.

In summary, waterlogging stress has become a major constraint for maize production. Here, we performed BSR-seq, and identified 18 high-probability SNPs and six candidate genes in response to waterlogging stress. The isolation, functional study and analysis of molecular mechanism of these candidate genes will be the focus in future research, which will add to the knowledge of waterlogging tolerance in maize.

## Author Contributions

HD and YX designed the study. JZ and HS performed maize waterlogging treatment and analyzed growth traits. JZ isolated total RNA from roots of maize, and performed qRT-PCR. XT, MH, BZ, and SW analyzed the phenotype data. HW, SD, and AL analyze the RNA-seq data. HD wrote the paper. All authors read and approved the final manuscript.

## Conflict of Interest Statement

The authors declare that the research was conducted in the absence of any commercial or financial relationships that could be construed as a potential conflict of interest.

## Footnotes

1 http://www.yeastgenome.org/help/analyze/go-term-finder

2 http://www.yeastgenome.org/help/analyze/go-term-finder

## Supplementary Material

FIGURE S1 The phenotypes of tolerant and sensitive inbred lines under waterlogging for 8 days or normal condition. The seedlings at three-leaf stage were subjected to waterlogging treatment for 8 days. **(A,D)** CML165; **(B,E)** DTMA217; **(C,F)** DTMA26; **(G,J)** DTMA280; **(H,K)** DTMA237; **(I,L)** DTMA103. N, means normal condition; W, means waterlogging treatment for 8 days.

TABLE S1 Primers used in this study.

TABLE S2 A total of 122 overlapped DEGs between tolerant and sensitive pool under waterlogging and normal condition.

TABLE S3 The DEGs in various GO items.

TABLE S4 A total of 114,580 high-confidence SNPs between the tolerant and sensitive pools under normal condition.

TABLE S5 A total of 114,464 high-confidence SNPs between the tolerant and sensitive pools under waterlogging.

TABLE S6 A total of 165 SNPs with high probabilities (>0.9) between the tolerant and sensitive pools under normal condition.

TABLE S7 A total of 128 SNPs with high probabilities (>0.9) between the tolerant and sensitive pools under waterlogging.

TABLE S8 The putative function of 18 genes with high-probability.
