# Supplementary figures and images for: Bulked Segregant RNA-seq Reveals Differential Expression and SNPs of Candidate Genes Associated with Waterlogging Tolerance in Maize
Source: Front Plant Sci. 2017 Jun 14;8:1022. doi: 10.3389/fpls.2017.01022 (PMC5470080; doi:10.3389/fpls.2017.01022)

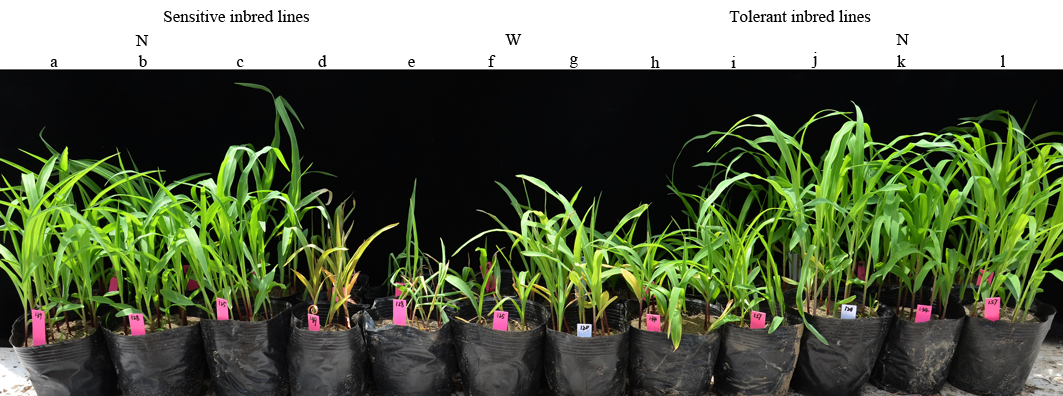

Supplement: FIGURE S1 — The phenotypes of tolerant and sensitive inbred lines under waterlogging for 8 days or normal condition. The seedlings at three-leaf stage were subjected to waterlogging treatment for 8 days. (A,D) CML165; (B,E) DTMA217; (C,F) DTMA26; (G,J) DTMA280; (H,K) DTMA237; (I,L) DTMA103. N, means normal condition; W, means waterlogging treatment for 8 days. [file Image_1.TIF]
